# Complete genome sequence of *Conexibacter woesei* type strain (ID131577^T^)

**DOI:** 10.4056/sigs.751339

**Published:** 2010-03-30

**Authors:** Rüdiger Pukall, Alla Lapidus, Tijana Glavina Del Rio, Alex Copeland, Hope Tice, Jan-Fang Cheng, Susan Lucas, Feng Chen, Matt Nolan, David Bruce, Lynne Goodwin, Sam Pitluck, Konstantinos Mavromatis, Natalia Ivanova, Galina Ovchinnikova, Amrita Pati, Amy Chen, Krishna Palaniappan, Miriam Land, Loren Hauser, Yun-Juan Chang, Cynthia D. Jeffries, Patrick Chain, Linda Meincke, David Sims, Thomas Brettin, John C. Detter, Manfred Rohde, Markus Göker, Jim Bristow, Jonathan A. Eisen, Victor Markowitz, Nikos C. Kyrpides, Hans-Peter Klenk, Philip Hugenholtz

**Affiliations:** 1 DSMZ – German Collection of Microorganisms and Cell Cultures GmbH, Braunschweig, Germany; 2 DOE Joint Genome Institute, Walnut Creek, California, USA; 3 Los Alamos National Laboratory, Bioscience Division, Los Alamos, New Mexico, USA; 4 Biological Data Management and Technology Center, Lawrence Berkeley National Laboratory, Berkeley, California, USA; 5 Oak Ridge National Laboratory, Oak Ridge, Tennessee, USA; 6 Lawrence Livermore National Laboratory, Livermore, California, USA; 7 HZI – Helmholtz Centre for Infection Research, Braunschweig, Germany; 8 University of California Davis Genome Center, Davis, California, USA

**Keywords:** aerobic, short rods, forest soil, *Solirubrobacterales*, *Conexibacteraceae*, GEBA

## Abstract

The genus *Conexibacter* (Monciardini *et al*. 2003) represents the type genus of the family *Conexibacteraceae* (Stackebrandt 2005, emend. Zhi *et al*. 2009) with *Conexibacter woesei* as the type species of the genus. *C. woesei* is a representative of a deep evolutionary line of descent within the class *Actinobacteria*. Strain ID131577^T^ was originally isolated from temperate forest soil in Gerenzano (Italy). Cells are small, short rods that are motile by peritrichous flagella. They may form aggregates after a longer period of growth and, then as a typical characteristic, an undulate structure is formed by self-aggregation of flagella with entangled bacterial cells. Here we describe the features of the organism, together with the complete sequence and annotation. The 6,359,369 bp long genome of *C. woesei* contains 5,950 protein-coding and 48 RNA genes and is part of the *** G****enomic* *** E****ncyclopedia of* *** B****acteria and* *** A****rchaea * project.

## Introduction

Strain ID131577^T^ (= DSM 14684 = JCM 11494) is the type strain of the species *Conexibacter woesei*, which is the type species of the genus *Conexibacter*. Strain ID131577^T^ was originally enriched from a soil sample used for isolation of filamentous actinomycetes and was first detected as a contaminant of a *Dactylosporangium* colony [1]. Based on 16S rRNA gene sequence analysis and the composition of their signature oligonucleotides, the strain was subsequently assigned to the subclass *Rubrobacteridae* within the class *Actinobacteria* [2]. Stackebrandt first placed the species *C. woesei* to the order *Rubrobacterales* [3,4]. With the description of *Patulibacter americanus* [5], the new order *Solirubrobacterales* was defined, again by the presence of 16S rRNA gene sequence signature oligonucleotides. The order *Solirubrobacterales* presently embraces the three families *Solirubrobacteraceae*, *Patulibacteraceae*, and *Conexibacteraceae* [5]; an emended description of the family *Conexibacteraceae* was published recently by Zhi *et al.* 2009 [6]. Several distantly related uncultured bacterial clones with less than 97% 16S rRNA gene sequence similarity to *C*. *woesei* were detected in various environmental habitats such as soil [7,8]; (EU223949, GQ366411), soil crusts [9], Fe-nodules of quaternary sediments in Japan [10], sediment (FN423884), rhizosphere [11], acidic *Sphagnum* peat bog [12], fleece rot (DQ221822), and salmonid gill [13]. *Conexibacter* related strains may also act as opportunistic pathogens as described in a few reports in which uncultured bacterial relatives were detected in bronchoalveolar fluid of a child with cystic fibrosis [14], or as enriched participants of showerhead biofilms [15]. Here we present a summary classification and a set of features for C. *woesei* ID131577^T^, together with the description of the complete genomic sequencing and annotation.

## Classification and features

**Figure 1:**
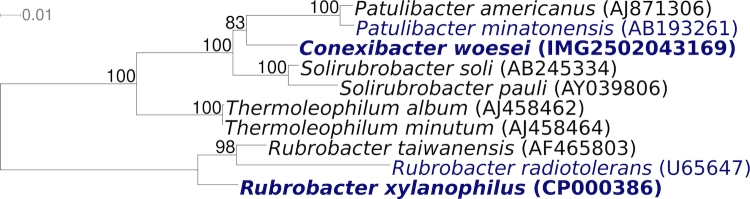
Phylogenetic tree highlighting the position of *C. woesei* ID131577^T^ relative to the other genera included in the subclass *Rubrobacteridae*. The tree was inferred from 1,429 aligned characters [16,17] of the 16S rRNA gene sequence under the maximum likelihood criterion [18] and rooted with the order *Rubrobacterales*. The branches are scaled in terms of the expected number of substitutions per site. Numbers above branches are support values from 1,000 bootstrap replicates if larger than 60%. Lineages with type strain genome sequencing projects registered in GOLD [19] are shown in blue, published genomes in bold.

Figure 1 shows the phylogenetic neighborhood for *C. woesei* ID131577^T^ in a 16S rRNA based tree. The single 16S rRNA gene sequence in the genome of *C. woesei* ID131577^T^ is 1,536 bp long. The previously published 16S rRNA sequence (AJ440237) covered 1,470 bp only, but is identical to the genome-derived sequence in that stretch.

**Figure 2:**
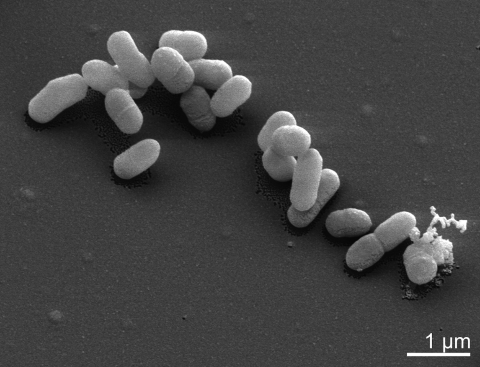
Scanning electron micrograph of *C. woesei* ID131577^T^

*C. woesei* is a Gram-positive, aerobic and non-sporulating bacterium, and forms small rods up to 1.2 µm in length (Table 1 and Figure 2). The strain is able to grow on complex media like TSA, BHI, Todd-Hewitt as well as on ISP2, ISP3 or R2A agar [1]. Growth occurs at pH 7-7.5 between 28 and 37°C. Catalase and oxidase activity is present and nitrate is reduced to nitrite. The strain is able to hydrolyze gelatin and esculin, but urea is not decomposed. Preferred substrates for utilization, as tested with the BiOLOG system, are L-arabinose, D-ribose, D-xylose, glycerol, acetic acid, pyruvic acid, propionic acid, α-ketovaleric acid, and ß-hydroxybutyric acid [1]. The strain is susceptible to amikacin, gentamicin, nitrofurantoin, polymyxin B, novobiocin and teichoplanin [1].

**Table 1:** Classification and general features of *C. woesei* ID131577^T^ according to the MIGS recommendations [20]

**MIGS ID**	**Property**	**Term**	**Evidence code**
	Current classification	Domain *Bacteria*	TAS [21]
		Phylum *Actinobacteria*	TAS [22]
		Class *Actinobacteria*	TAS [2]
		Subclass *Rubrobacteridae*	TAS [2]
		Order *Solirubrobacterales*	TAS [5]
		Family *Conexibacteraceae*	TAS [3,6]
		Genus *Conexibacter*	TAS [1]
		Species *Conexibacter woesei*	TAS [1]
		Type strain ID131577	TAS [1]
	Gram stain	positive	TAS [1]
	Cell shape	short rods	TAS [1]
	Motility	long, peritrichous flagella	TAS [1]
	Sporulation	unknown	NAS
	Temperature range	28°C-37°C	TAS [1]
	Optimum temperature	unknown	
	Salinity	< 2%	TAS [1]
MIGS-22	Oxygen requirement	strictly aerobic	TAS [1]
	Carbon source	saccharolytic	TAS [1]
	Energy source	carbohydrates	TAS [1]
MIGS-6	Habitat	soil	TAS [1]
MIGS-15	Biotic relationship	free living	NAS
MIGS-14	Pathogenicity	opportunistic	NAS
	Biosafety level	1	TAS [23]
	Isolation	soil	TAS [1]
MIGS-4	Geographic location	Gerenzano, Italy	TAS [1]
MIGS-5	Sample collection time		TAS [1]
MIGS-4.1 MIGS-4.2	Latitude Longitude	45.640 9.002	NAS
MIGS-4.3	Depth	not reported	
MIGS-4.4	Altitude	not reported	

Evidence codes - IDA: Inferred from Direct Assay (first time in publication); TAS: Traceable Author Statement (i.e., a direct report exists in the literature); NAS: Non-traceable Author Statement (i.e., not directly observed for the living, isolated sample, but based on a generally accepted property for the species, or anecdotal evidence). These evidence codes are from of the Gene Ontology project [24]. If the evidence code is IDA, then the property was directly observed by one of the authors or an expert mentioned in the acknowledgements.

### Chemotaxonomy

*C. woesei* possesses a peptidoglycan type of A1γ, based on mesoA_2_pm. Meso-diaminopimelic acid is the diagnostic amino acid at position 3 of the peptidoglycan for all members of the order *Solirubrobacterales*, whereas members of the *Rubrobacterales* are characterized by L-Lys as the diamino acid at position 3 (peptidoglycan type A3α, L-Lys ← L-Ala). The tetrahydrogenated menaquinone MK-7(H_4_) was detected as the major component in *C. woesei* and *Solirubrobacter pauli* [1,25]. This is a remarkable feature, because MK-7(H_4_) if detectable in bacteria, has previously been reported as a minor component only. The main polar lipid was identified by two-dimensional TLC as phosphatidylinositol. Oleic acid (C_18:1ω9c_), 14-methyl-pentadecanoic acid (i-C_16:0_), hexadecanoic acid (C_16:0_) and ω6c-heptadecenoic acid (C_17:1ω6c_) constituted the major cellular fatty acids [1]. Mycolic acids are absent.

## Genome sequencing and annotation

### Genome project history

This organism was selected for sequencing on the basis of its phylogenetic position, and is part of the *** G****enomic* *** E****ncyclopedia of* *** B****acteria and* *** A****rchaea * project [26]. The genome project is deposited in the Genome OnLine Database [19] and the complete genome sequence is deposited in GenBank. Sequencing, finishing and annotation were performed by the DOE Joint Genome Institute (JGI). A summary of the project information is shown in Table 2.

**Table 2:** Genome sequencing project information

**MIGS ID**	**Property**	**Term**
MIGS-31	Finishing quality	Finished
MIGS-28	Libraries used	Two Sanger libraries: 8kb pMCL200 and fosmid pcc1Fos. One 454 pyrosequence standard library
MIGS-29	Sequencing platforms	ABI3730, 454 GS FLX,
MIGS-31.2	Sequencing coverage	10.0× Sanger; 19.15× pyrosequence
MIGS-30	Assemblers	Newbler version 1.1.02.15, phrap
MIGS-32	Gene calling method	Prodigal, GenePRIMP
	INSDC ID	CP001854
	Genbank Date of Release	January 15, 2010
	GOLD ID	Gc01185
	NCBI project ID	20745
	Database: IMG-GEBA	2501939629
MIGS-13	Source material identifier	DSM 14684
	Project relevance	Tree of Life, GEBA

### Growth conditions and DNA isolation

*C. woesei* ID131577^T^, DSM 14684, was grown in DSMZ medium 92 [27] at 28°C. DNA was isolated from 0.5 to 1 g of cell paste using Qiagen Genomic 500 DNA Kit (Qiagen, Hilden, Germany) with cell lysis modification st/L [26] and over night incubation at 35°C.

### Genome sequencing and assembly

The genome was sequenced using a combination of Sanger and 454 sequencing platforms. All general aspects of library construction and sequencing can be found at http://www.jgi.doe.gov/. 454 Pyrosequencing reads were assembled using the Newbler assembler version 1.1.02.15 (Roche). Large Newbler contigs were broken into 6,955 overlapping fragments of 1,000 bp and entered into assembly as pseudo-reads. The sequences were assigned quality scores based on Newbler consensus q-scores with modifications to account for overlap redundancy and to adjust inflated q-scores. A hybrid 454/Sanger assembly was made using the parallel phrap assembler (High Performance Software, LLC). Possible mis-assemblies were corrected with Dupfinisher [28] or transposon bombing of bridging clones (Epicentre Biotechnologies, Madison, WI). Gaps between contigs were closed by editing in Consed, custom primer walk or PCR amplification. A total of 1,608 Sanger finishing reads were produced to close gaps, to resolve repetitive regions, and to raise the quality of the finished sequence. The error rate of the completed genome sequence is less than 1 in 100,000. Together all sequence types provided 29.15× coverage of the genome. The final assembly contains 79,136 Sanger and 580,261 pyrosequence reads.

### Genome annotation

Genes were identified using Prodigal [29] as part of the Oak Ridge National Laboratory genome annotation pipeline, followed by a round of manual curation using the JGI GenePRIMP pipeline [30]. The predicted CDSs were translated and used to search the National Center for Biotechnology Information (NCBI) nonredundant database, UniProt, TIGRFam, Pfam, PRIAM, KEGG, COG, and InterPro databases. Additional gene prediction analysis and manual functional annotation was performed within the Integrated Microbial Genomes Expert Review (IMG-ER) platform [31].

## Genome properties

**Figure 3:**
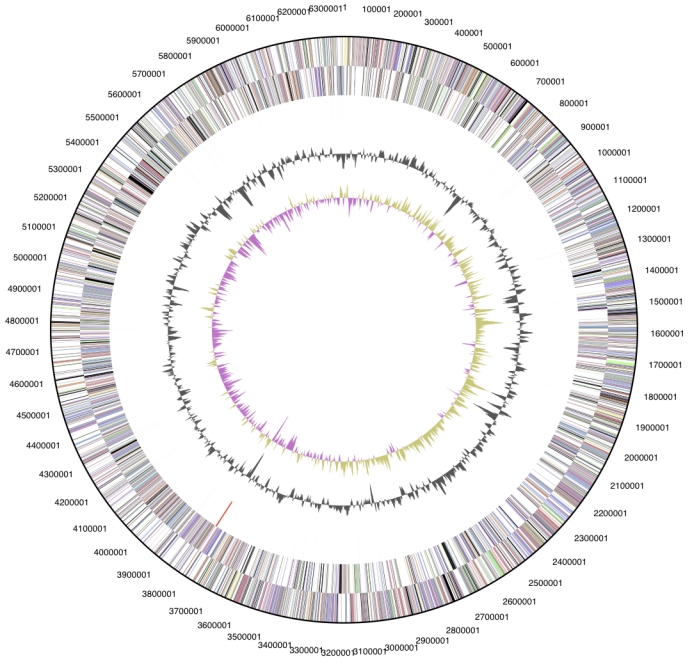
Graphical circular map of the chromosome. From outside to the center: Genes on forward strand (color by COG categories), Genes on reverse strand (color by COG categories), RNA genes (tRNAs green, rRNAs red, other RNAs black), GC content, GC skew.

The genome consists of a 6,359,369 bp long chromosome. Of the 5,998 genes predicted, 5,950 were protein-coding genes, and 48 RNAs; 36 pseudogenes were also identified (Table 3 and Figure 3). The majority of the protein-coding genes (74.5%) were assigned with a putative function while those remaining were annotated as hypothetical proteins. The distribution of genes into COGs functional categories is presented in Table 4.

**Table 3:** Genome Statistics

**Attribute**	**Value**	**% of Total**
Genome size (bp)	6,359,369	100.00%
DNA coding region (bp)	6,001,841	94.38%
DNA G+C content (bp)	4,625,387	72.73%
Number of replicons	1	
Extrachromosomal elements	0	
Total genes	5,998	100.00%
RNA genes	48	0.80%
rRNA operons	1	
Protein-coding genes	5,950	99.20%
Pseudo genes	36	0.61%
Genes with function prediction	4,466	74.46%
Genes in paralog clusters	1,697	28.52%
Genes assigned to COGs	4,419	74.27%
Genes assigned Pfam domains	4,500	75.03%
Genes with signal peptides	1,632	27.43%
Genes with transmembrane helices	1,444	24.27%
CRISPR repeats	0	0

**Table 4:** Number of genes associated with the general COG functional categories

**Code**	**value**	**%age**	**Description**
J	197	3.9	Translation, ribosomal structure and biogenesis
A	3	0.1	RNA processing and modification
K	550	10.8	Transcription
L	120	2.3	Replication, recombination and repair
B	2	0.0	Chromatin structure and dynamics
D	30	0.6	Cell cycle control, cell division, chromosome partitioning
Y	0	0.0	Nuclear structure
V	89	1.7	Defense mechanisms
T	287	5.6	Signal transduction mechanisms
M	241	4.7	Cell wall/membrane biogenesis
N	57	1.1	Cell motility
Z	1	0.0	Cytoskeleton
W	0	0.0	Extracellular structures
U	44	0.9	Intracellular trafficking and secretion
O	131	2.6	Posttranslational modification, protein turnover, chaperones
C	325	6.4	Energy production and conversion
G	396	7.8	Carbohydrate transport and metabolism
E	566	11.1	Amino acid transport and metabolism
F	84	1.6	Nucleotide transport and metabolism
H	186	3.6	Coenzyme transport and metabolism
I	252	4.9	Lipid transport and metabolism
P	301	5.9	Inorganic ion transport and metabolism
Q	220	4.3	Secondary metabolites biosynthesis, transport and catabolism
R	705	13.8	General function prediction only
S	323	6.3	Function unknown
-	1,579	26.3	Not in COGs
